# Hodgkin–Huxley revisited: reparametrization and identifiability analysis of the classic action potential model with approximate Bayesian methods

**DOI:** 10.1098/rsos.150499

**Published:** 2015-12-23

**Authors:** Aidan C. Daly, David J. Gavaghan, Chris Holmes, Jonathan Cooper

**Affiliations:** 1 Department of Computer Science, University of Oxford, Oxford, UK; 2 Department of Statistics, University of Oxford, Oxford, UK

**Keywords:** Hodgkin Huxley, approximate Bayesian computation, identifiability, cardiac cell modelling, functional curation, parameter fitting

## Abstract

As cardiac cell models become increasingly complex, a correspondingly complex ‘genealogy’ of inherited parameter values has also emerged. The result has been the loss of a direct link between model parameters and experimental data, limiting both reproducibility and the ability to re-fit to new data. We examine the ability of approximate Bayesian computation (ABC) to infer parameter distributions in the seminal action potential model of Hodgkin and Huxley, for which an immediate and documented connection to experimental results exists. The ability of ABC to produce tight posteriors around the reported values for the gating rates of sodium and potassium ion channels validates the precision of this early work, while the highly variable posteriors around certain voltage dependency parameters suggests that voltage clamp experiments alone are insufficient to constrain the full model. Despite this, Hodgkin and Huxley's estimates are shown to be competitive with those produced by ABC, and the variable behaviour of posterior parametrized models under complex voltage protocols suggests that with additional data the model could be fully constrained. This work will provide the starting point for a full identifiability analysis of commonly used cardiac models, as well as a template for informative, data-driven parametrization of newly proposed models.

## 1. Introduction

Cardiac cells have been one of the most popular targets of mathematical biological modelling since the field's inception in the late 1940s. This is partly owing to the difficulty of performing clinical studies on human hearts, as well as the high species dependence of electropysiological properties such as action potential (AP) shape, duration (APD) and restitution properties that limit the applicability of studies in model systems [1]. In the face of this paucity of data, simulations from these models are used as a primary level of inference for the impact of mutations or drugs on cellular- or organ-level properties [2–4], and can also inform the design of future experiments. Analysis of the ways in which simulations fail to reconstruct experimental recordings often leads to a better understanding of the data needed to correct the model in the next iteration [5].

One of the most influential and enduring cellular models is the Hodgkin–Huxley AP model [6]. Though the model was constructed for the squid giant axon, the experimental set-up and formulation of model components set a standard that was adopted by many cardiac models, and persists to this day. From their original voltage clamp experimental data [7], the authors determined that the ionic conductances were best explained by a system of nonlinear ordinary differential equations (ODEs) which, unbeknown to them, can describe by direct biological analogy the opening and closing dynamics of membrane ion channels. These equations ((2.6)–(2.16) in §2) serve as the foundation for the so-called ‘Hodgkin–Huxley style’ formulation for ion channel modelling, where all channel transitions are modelled by ODEs. To this day, many cardiac models incorporate this formulation for modelling ion channels when performing large-scale simulations owing to its simplicity when compared to a more general Markov formulation [8].

At the time, parametrizing the Hodgkin–Huxley model required meticulously matching hand-drawn curves to experimental data. Despite the advances in model fitting techniques from these pen and paper methods, however, a commensurate increase in cardiac model complexity has made it difficult to obtain enough independent data to fully employ such methods. Advances in recording techniques such as the patch clamp experiment [9] have driven the inclusion of more ion channels and intracellular dynamics with the intention of forming a more complete representation of the true cellular environment, increasing the amount of experimentation required to fully observe a system (see [5,10] for a more complete discussion of the history of cardiac modelling). The modelling of ion channel kinetics in particular has posed a problem, as most cardiac data are macroscopic, and isolation/expression systems to study single channels can alter their native behaviour [4]. In the face of these difficulties in obtaining data for modelling experiments, many modellers borrow or adapt parameter values, or even entire model subunits, from previous models, which were often constructed for different experimental systems [11,12].

While much effort is taken to adjust model components for differences in species and/or experimental conditions, as well as to maintain certain macroscopic properties such as AP shape, comparative analyses have shown discrepancies in behaviour between sequentially fit models purporting to represent identical systems [13–15]. This indicates a fragility in these models that limits our confidence in their predictive power outside the range of their validated behaviour. While some of these discrepancies may be attributed to biological variability, poor documentation or justification of parameter inheritance has made it increasingly difficult to link parametrized components to original data that might capture or explain this variation [14,16], leading to a weakening link to the mechanistic underpinning of some of the more complex models being proposed today.

This devolving link between experimental data and parametrized models makes the application of modelling techniques which provide posterior distributions over parameters (rather than single optimal values) very difficult. Paradoxically, therefore, one of the most mature areas of systems biology has perhaps benefited least from the application of the advanced methods of parameter fitting and model selection that have been applied to more recently developed areas [17,18]. In light of this problem, the original Hodgkin–Huxley model becomes an ideal case study for illustrating and assessing the utility of the application of modern inference techniques to cardiac AP models based on the Hodgkin–Huxley formulation. All data used by Hodgkin and Huxley were originally and consistently produced by the authors, and therefore provide the ideal test-bed for exploring the relationship between parameter values and experimental data for these types of models.

Application of these new techniques also allows us to explore the issue of model identifiability for Hodgkin–Huxley type models, that is, the degree to which the ‘optimal’ parameters for the model are unique. Published models are underpinned by an assumption that the given parametrization is unique for the data that were used to fit them. This assumption is inherent in the use of classical fitting methods such as least-squares regression, which output only a single optimal point in parameter space. We know, however, that such a unique optimum is unlikely to exist in biological systems, as both inter- and intracellular variations may be reflected by variations in the biophysical parameters of the model. Therefore, by examining posterior distributions over model parameters, we can quantify and characterize model uncertainty, which can either inform us as to the ability of our data to constrain the model or the degree of biological variability in the system, as we outline below.

If a cellular model were to have a non-unique optimal parametrization, with either several or infinitely many equally likely possibilities, the model may be deemed ‘unidentifiable’. Unidentifiability can be divided into two types: structural unidentifiability, where the model is overly complex to describe the system (this if often also called over-parametrization), and practical unidentifiability, where there are not enough data to fully constrain the model [19]. Distinguishing between the two types of unidentifiability can be important for experimental design. Pinpointing a structural unidentifiability, which is characterized by a functional relationship between several parameters (and thus infinitely many equally optimal parametrizations), may be a cause for concern, and prompt changes in model formulation before attempting further experimentation. However, if there is a high degree of prior faith in the model formulation, this may instead suggest a biologically important redundancy in the system characterized by the functional relationship between biophysical parameters. Practical unidentifiability, on the other hand, may inform how additional experimental data might be collected in an effort to further constrain the model [19,20], or may simply be a representation of inherent variability of the biophysical parameters in the experimental system.

While there is no universally accepted automated means to assess identifiability [21], many different methods, such as parameter sensitivity analysis [2,22,23] and analysis of curvature of an objective function [24,25], have been applied to cardiac cell models, and there now exist documented concerns for model identifiability in both widely used Hodgkin–Huxley and Markov style models under common experimental protocols [21,24,26].

When experimental data are available to us, however, an appropriately chosen Bayesian method for parameter fitting can also be used to assess the identifiability of a model. Bayesian inference involves calculating a full posterior probability distribution around the model parameters, the shape and width of which can inform the modeller as to the degree of variation about the optimal parameter value. A wide, flat posterior on a parameter, for example, indicates a large number of equally optimal values, which suggests that the parameter may be unidentifiable [27]. We expect variation arising from natural biological variation to manifest itself as a well-formed distribution, the statistical properties of which can be used to draw conclusion as to the nature or source of such variation, while variation arising from insufficient data would be more erratic, related to noise in the experimental data or random choices made by the algorithm when the observed data does not provide much information about a portion of the model dynamics. Because only further experimentation can firmly distinguish the two (inherent biological variation should remain unaffected by the inclusion of additional data), the automated design of ‘optimal’ experiments to attempt to reduce uncertainty is an area of current interest [28].

Because fully Bayesian inference involves potentially intractable integrals, much of the research in this area has been into methods to speed up or reasonably approximate calculation of the posterior [29,17]. Approximate Bayesian computation (ABC), first employed for parameter selection by Tavare *et al.* [30], gives a means to generate a population of solutions by repeatedly sampling from a prior distribution over model parameters and accepting draws based on an objective function evaluation. Bayesian inference also gives us the ability to include data from multiple sources in a principled manner, often weighted by the degree of noise present or prior knowledge as to its relative significance. This allows us to include data from multiple repetitions of an experiment at once, allowing both mean behaviour and variability of the experiment to inform posterior parameter distributions.

We aim to use ABC to investigate the identifiability of the Hodgkin and Huxley model by re-fitting both the basic (equations (2.6)–(2.10)) and expanded (equations (2.11)–(2.16)) form to the authors' original published data. We will assess the identifiability of the model by examining the width of the resulting posterior estimates for all parameters in each model and attempt to classify unidentifiabilities as structural or practical by examining model output fluctuations and parameter correlations across the posterior. Finally, we will examine the potential of using more complex voltage protocols proposed by the original authors to further constrain the voltage-dependent model. This analysis allows not only the assessment of the ability of the original manual parameter fitting methods to match modern automated techniques, but also serves as an example for the implementation of informative parameter estimation techniques in cardiac modelling.

We have released code implementing these techniques so that modellers can apply these Bayesian parameter fitting techniques to their own systems of study, providing an unambiguous link between components of published models and experimental data. This code was built on the functional curation extension [31,32] to the Chaste cardiac simulation library [33], which allows the specification of stand-alone simulation protocols that can be applied to a range of *in silico* models, in order to ease the mapping between simulated and real data. The adoption of such standards would allow modellers to quantify their certainty in a published model given the experimental data employed, and thus allow for the informed adoption of a model or its components by their peers.

In §2, we present both forms of the Hodgkin–Huxley model in full detail. In §3, we discuss the details of our implementation of ABC, as well as our use of functional curation. In §4, we present the results of our ABC re-fitting experiments on the two forms of the Hodgkin–Huxley model, and analyse the properties of the resulting posteriors. In §5, we discuss the implications of the identifiability analysis for potential improvements to the design of experiments used to fit the model, and finally conclude in §6.

## 2. The Hodgkin–Huxley model

Hodgkin and Huxley treated the squid axon as an electrical circuit, with current across the membrane being carried by a capacitor or by one of three ionic currents: *I*_*K*_, the current carried by potassium ions, *I*_*Na*_, the current carried by sodium ions, and *I*_*l*_, a catch-all leakage current. Thus, the fundamental equations for simulating membrane potential changes were as follows:

2.1 $$\begin{array}{rl} I & =C_{M}\frac{dV}{dt}+I_{i}, \end{array}$$

2.2 $$\begin{array}{rl} I_{i} & =I_{\mathrm{Na}}+I_{K}+I_{l}, \end{array}$$

2.3 $$\begin{array}{rl} I_{\mathrm{Na}} & =g_{\mathrm{Na}}(V-V_{\mathrm{Na}}), \end{array}$$

2.4 $$\begin{array}{rl} I_{K} & =g_{K}(V-V_{K}) \end{array}$$

2.5 $$\begin{array}{rl} \mathrm{and}\;I_{l} & =g_{l}(V-V_{l}), \end{array}$$

where *dV* /*dt* is the rate of change in membrane potential, *C*_*M*_ is the membrane capacitance, and *I*_*i*_ is the sum of the three ionic currents. For each ionic current *x*, *V*_*x*_ represents the reversal potential (the membrane potential at which there is no net flow of that ion) and *g*_*x*_ is the membrane conductance per unit area for that ion.

The ionic conductances, with the exception of the leakage current *g*_*l*_ which was assumed constant, were theorized to be explained by the following ODEs:

**Table:** 

conductance type	equations
potassium	$$\begin{array}{rlr} g_{K} & ={\bar{g}}_{K}n^{4}, & \;2.6\\ \frac{dn}{dt} & =\alpha_{n}(1-n)-\beta_{n}n, & \;2.7 \end{array}$$
sodium	$$\begin{array}{rlr} g_{\mathrm{Na}} & ={\bar{g}}_{\mathrm{Na}}m^{3}h, & \;2.8\\ \frac{dm}{dt} & =\alpha_{m}(1-m)-\beta_{m}m, & \;2.9\\ \frac{dh}{dt} & =\alpha_{h}(1-h)-\beta_{h}h, & \;2.10 \end{array}$$
**free parameters:***α*_*n*_, *β*_*n*_, *α*_*m*_, *β*_*m*_, *α*_*h*_, *β*_*h*_	

where ${\bar{g}}_{K}$ and ${\bar{g}}_{\mathrm{Na}}$ describe the maximum ionic conductances. The dimensionless variables *m*, *n* and *h* describe the open probability of ion channels (and thus the expected fraction of the full ionic conductance across the membrane at a given time and voltage), and the *α* and *β* terms are rate constants.

After choosing rate parameters *α* and *β* for a variety of clamped voltages, Hodgkin and Huxley empirically proposed functions that could explain the voltage dependency of these parameters, yielding the following equations:

**Table:** 

conductance type	equations
potassium	$$\begin{array}{rlr} {\hat{\alpha }}_{n}(V) & =\frac{0.01(V+10)}{\exp\left(\frac{V+10}{10}\right)-1}, & \;2.11\\ {\hat{\beta }}_{n}(V) & =0.125\exp(V/80), & \;2.12 \end{array}$$
sodium	$$\begin{array}{rlr} {\hat{\alpha }}_{m}(V) & =\frac{0.1(V+25)}{\exp\left(\frac{V+25}{10}\right)-1}, & \;2.13\\ {\hat{\beta }}_{m}(V) & =4\exp(V/18), & \;2.14\\ {\hat{\alpha }}_{h}(V) & =0.07\exp(V/20), & \;2.15\\ {\hat{\beta }}_{h}(V) & =\frac{1}{\exp\left(\frac{V+30}{10}\right)+1}. & \;2.16 \end{array}$$

## 3. Methods

All Hodgkin–Huxley conductance data were obtained from figs 3 and 6 digitized from the original publication using Plot Digitizer (http://plotdigitizer.sourceforge.net). Plot *L* of the potassium conductance data (figure 3, depolarization of −6 mV) was eliminated owing to the lack of a reliable scale on the *y*-axis.

### 3.1 Model simulations

All simulations for ABC were carried out using the current development version of the Python implementation of functional curation [33,31] (https://chaste.cs.ox.ac.uk/trac/browser/projects/FunctionalCuration).

#### 3.1.1 Hodgkin–Huxley CellML model

A CellML [34] model file for Hodgkin–Huxley was annotated with metadata tags to allow the adjustment of the *α* and *β* parameters (equations (2.7), (2.9) and (2.10)) by the fitting algorithm. Similarly, the constants in equations (2.11)–(2.16) were replaced with free, externally adjustable, variables as detailed in equations (3.1)–(3.6), giving five free parameters for the voltage-dependent potassium conductance *g*_K_ and nine free parameters for the voltage-dependent sodium conductance *g*_Na_.

#### 3.1.2 Voltage clamp protocol

A functional curation protocol, which details the external stimuli applied to a corresponding *in silico* model (parsed from a CellML file), was written to replicate the voltage clamp experiments in figs 3 and 6 of the original publication. Initial conditions for the ODEs and maximum conductance values were set as reported in tables 1 and 2 of the same [6]. Each voltage clamp experiment was carried out by redefining the model's membrane voltage to a fixed value from within the protocol, then simulating the model over a 12 ms time course from the initial state, recording the two ionic conductance values every 0.1 ms.

**Table 1.** Specification of prior and kernel distributions employed by ABC for each of the parameters in the simplified (equations (2.6)–(2.10)) and expanded (equations (3.1)–(3.6)) Hodgkin–Huxley models.

variable(s)	prior distribution	kernel distribution
*α*_*n*_, *β*_*n*_	uniform(0, 1)	N(0,0.1)
*α*_*m*_, *β*_*m*_, *α*_*h*_, *β*_*h*_	uniform(0, 10)	N(0,1)
*k*_*α*_*n*_1_, *k*_*β*_*n*_1_, *k*_*α*_*m*_1_, *k*_*α*_*h*_1_	uniform(0, 1)	N(0,0.1)
*k*_*β*_*m*_1_	uniform(0, 10)	N(0,1)
*k*_*α*_*n*_3_, *k*_*β*_*n*_2_, *k*_*α*_*m*_3_, *k*_*β*_*m*_2_, *k*_*α*_*h*_2_, *k*_*β*_*h*_2_	uniform(1, 100)	N(10)
*k*_*α*_*n*_2_, *k*_*α*_*m*_2_, *k*_*β*_*h*_1_	uniform(0, 100)	N(10)

**Table 2.** Summary statistics of ABC posterior estimates for voltage dependency parameters of potassium conductance gating rates. (Parameter names in the leftmost column refer to the corresponding labels in equations (3.1)–(3.2). ‘Mean’ and ‘variance’ columns indicate the summary statistics used to describe the final posterior estimate of ABC, while ‘5th, 95th percentiles’ denote the points at which the posterior CDF records 5% and 95% density, respectively (analogous to 90% CI). The ‘RMSE’ row contains the value of the distance metric (equation (3.2)) attained by the reported parameters, as well as the minimum and maximum (bounded from above by the final ABC error constraint *ϵ*_*T*_) values attained by particles in the final posterior estimate.)

		ABC posterior estimates		
parameter	reported value	mean	variance	5th, 95th percentiles
*k*_*α*_*n*_1_	0.01	0.0128	4.29×10^−6^	0.0103, 0.0177
*k*_*α*_*n*_2_	10	44.8	162	27.1, 62.1
*k*_*α*_*n*_3_	10	30.7	48.2	18.7, 37.4
*k*_*β*_*n*_1_	0.125	0.177	3.14×10^−3^	0.0986, 0.253
*k*_*β*_*n*_2_	80	65.1	218	48.9, 95.2
RMSE	0.642	min 0.559, max (≤*ϵ*_*T*_) 0.794		

When fitting the limited six-parameter form of the model (equations (2.7), (2.9) and (2.10)), the rate parameters *α*_*n*_, *β*_*n*_, *α*_*m*_, *β*_*m*_, *α*_*h*_ and *β*_*h*_ are defined by the protocol to be constants, which are set by the fitting algorithm. When fitting the full 14-parameter voltage-dependent model, these six rate parameters are in turn parametrized according to equations (3.1)–(3.6), with the 14 free parameters set by the fitting algorithm:

**Table:** 

conductance type	equations
potassium	$$\begin{array}{rlr} \alpha_{n}\left(V\right) & =\frac{k_{\alpha_{n}1}\left(V+k_{\alpha_{n}2}\right)}{\exp\left(\frac{V+k_{\alpha_{n}2}}{k_{\alpha_{n}3}}\right)-1}, & \;3.1\\ \beta_{n}\left(V\right) & =k_{\beta_{n}1}\exp\left(V/k_{\beta_{n}2}\right), & \;3.2 \end{array}$$
sodium	$$\begin{array}{rlr} \alpha_{m}\left(V\right) & =\frac{k_{\alpha_{m}1}\left(V+k_{\alpha_{m}2}\right)}{\exp\left(\frac{V+k_{\alpha_{m}2}}{k_{\alpha_{m}3}}\right)-1}, & \;3.3\\ \beta_{m}\left(V\right) & =k_{\beta_{m}1}\exp\left(V/k_{\beta_{m}2}\right), & \;3.4\\ \alpha_{h}\left(V\right) & =k_{\alpha_{h}1}\exp\left(V/k_{\alpha_{h}2}\right), & \;3.5\\ \beta_{h}\left(V\right) & =\frac{1}{\exp\left(\frac{V+k_{\beta_{h}1}}{k_{\beta_{h}2}}\right)+1}. & \;3.6 \end{array}$$
**free parameters:***k*_*α*_*n*_1_, *k*_*α*_*n*_2_, *k*_*α*_*n*_3_, *k*_*β*_*n*_1_, *k*_*β*_*n*_2_,	
*k*_*α*_*m*_1_, *k*_*α*_*m*_2_, *k*_*α*_*m*_3_, *k*_*β*_*m*_1_, *k*_*β*_*m*_2_,	
*k*_*α*_*h*_1_, *k*_*α*_*h*_2_, *k*_*β*_*h*_1_, *k*_*β*_*h*_2_	

#### 3.1.3 Other functional curation protocols

**Figure 1.**
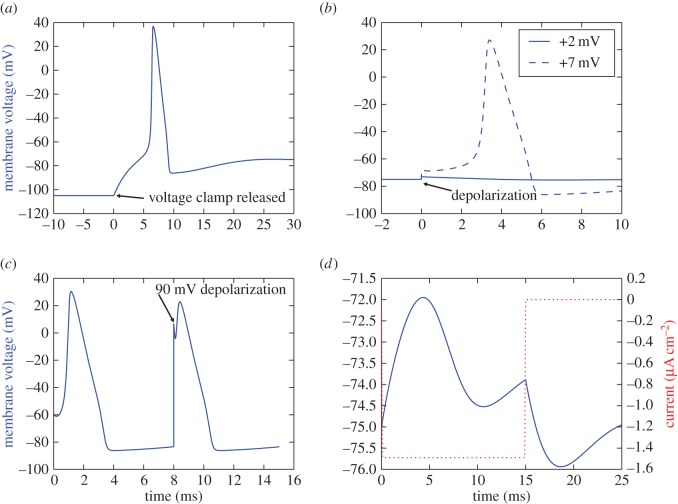
Stimulus-response representation of the four advanced voltage protocols proposed by Hodgkin and Huxley. Membrane voltage, the output quantity, is represented in blue, and in all figures is calculated with equations (2.11)–(2.16). In the anode break (*a*), threshold excitation (*b*) and positive phase depolarization (*c*) experiments, membrane voltage is manipulated directly as described in §iii. The graph representing the oscillation induction experiment (*d*) shows the induced stimulus current (dotted red) and the resulting fluctuation in membrane potential. In all graphs, the models have reached steady-state by time *t*=0 when stimulus is applied.

Functional curation protocols were written for the four more complex voltage protocols described by Hodgkin and Huxley in figs 20–23 of their original publication [6]. Each of these protocols accepted the 14 voltage dependency parameters from equations (3.1)–(3.6) as input and set the corresponding model variables to provide input values at the start of the simulation. Each protocol subjected the model to an initial 1000 ms time course simulation to reach steady state for the new parametrization, after which all state variables *n*, *m* and *h* were shown to have reached constant values. Graphical representations of all protocols can be found in figure 1.

*Anode break*. After reaching steady state, the model was subjected to a 200 ms voltage clamp to 30 mV below resting potential. This clamp was then released and the membrane voltage recorded every 0.1 ms for the duration of a 30 ms time course simulation (figure 1*a*). The reversible voltage clamp was attained by redefining *V* in the model interface of the protocol to be constant between 1000 and 1200 ms and to follow the original model definition at all other time intervals.

*Threshold excitation*. After reaching steady state, the model was subjected to a membrane depolarization of either −10 mV, 2 mV, 5 mV 6 mV, or 7 mV, with only the latter being sufficient to trigger an AP under reported values for gating rate voltage dependency parameters (figure 1*b*). The membrane voltage was then recorded every 0.01 ms for the duration of a 10 ms time course simulation.

*Positive phase depolarization*. After reaching steady state, the model was subjected to a 15 mV membrane depolarization followed by a 5 ms, 6 ms or 8 ms time course simulation. After this, the model was subjected to an additional 90 mV depolarization and the remainder of a 15 ms total time course simulation (figure 1*c*). Membrane voltage was recorded every 0.01 ms for the total duration of the 15 ms time course following the initial depolarization.

*Oscillation induction*. After reaching steady state, the membrane current of the model was clamped to −1.49 mA cm^−2^ for the duration of a 15 ms time course simulation. The clamp was then released and the model subjected to an additional 10 ms time course simulation, with membrane voltage being recorded every 0.1 ms for the entirety of the 25 ms time course (figure 1*d*). The reversible current clamp was attained by redefining the membrane stimulus current to be constant between 1000 and 1015 ms and to follow the original model definition at all other time intervals.

### 3.2 Approximate Bayesian computation

#### 3.2.1 Approximate Bayesian computation general settings and adaptive error shrinking

The simplest form of ABC is known as the rejection sampler. In this scheme, parameters are continually sampled from a specified prior distribution and used to simulate model output. Parameter sets that generate simulated output close to the experimental data are accepted and are added as ‘particles’ of a population of solutions that estimate the true posterior. Thus, for the rejection sampler, all that is required is the specification of prior parameter functions, a distance function between simulated and experimental output, and an acceptance tolerance for this function.

When the prior and posterior distributions of parameters differ greatly, however, this method is impractical, as very few samples from the prior are expected to be accepted. More sophisticated variants of ABC create a series of posterior estimates, each one drawing samples from the one before it (rather than the prior). The error tolerance is initially relaxed, leading to a high number of acceptances, but is gradually tightened between rounds of sampling. Such a scheme will smooth the difference between the prior and the posterior, sequentially narrowing the range of accepted parameter values. We employed such a variation of ABC described by Toni *et al.* as ‘sequential Monte Carlo’ (ABC-SMC), which generates a series of posterior estimates of fixed size as follows:

(i) draw *N* parameter vectors from the prior distribution *π*(***θ***) to form the initial posterior estimate ***θ***_0_. Each component particle $\theta_{0}^{\left(i\right)}$ (*i*∈[1,*N*]) of this initial estimate will be assigned an initial, uniform weight of $w_{0}^{\left(i\right)}=1/N$;
(ii) for each subsequent posterior estimate ***θ***_*t*_ (*t*∈[1,*T*]), update each particle $\theta_{t}^{\left(i\right)}$ (*i*∈[1,*N*]) in the following manner:
  — draw a particle from the previous posterior estimate ***θ***_*t*−1_ and slightly perturb the parameter values according to a (stochastic) function *K*(*θ*) to obtain θ*. Repeat this drawing and perturbation until θ* is legal (i.e. has non-zero likelihood under the original prior *π*(***θ***));
  — simulate model output y* under parameters θ*. If the distance function between the simulated data and the original data $D({\text{y}}^{*},{\text{y}}_{\mathrm{true}})$ is less than *ϵ*_*t*_<*ϵ*_*t*−1_, ACCEPT $\theta_{t}^{\left(i\right)}=\theta^{*}$. Otherwise, REJECT and repeat from the previous step;
  — set the weight $w_{t}^{\left(i\right)}$ of particle $\theta_{t}^{\left(i\right)}$ to $P(\theta_{t}^{\left(i\right)}|\theta_{t-1})$, the probability of obtaining the particle as a perturbed draw from the previous estimate. In order to calculate this probability, the kernel function *K*(*θ*) must either be reversible (*P*(*K*(*θ*)=θ*)=*P*(*K*(θ*)=*θ*)) or the so-called ‘backward kernel’ *K*^−1^ (*P*(*K*(*θ*)=θ*)=*P*(*K*^−1^(θ*)=*θ*)) must be provided; and
(iii) when all *N* particles are updated, normalize the weights **w**_*t*_ and begin the next iteration (t ← t + 1).

The choice of the so-called kernel function *K*(*θ*) is important, as the perturbations it induces to the draws promotes exploration around previously accepted parameter estimates with the intention of generating better ones. Too much deviation from the original values, however, may lead to erratic behaviour in the draws and lower the acceptance rate. Thus, a kernel function must balance between locality and exploration, and must take into account the scale of the model parameters in order to generate valid perturbations. The variance of the final posterior estimate can be used to quantify the identifiability of each parameter, or of the model as a whole.

The main drawback of ABC-SMC is the need to pick an appropriate ‘cooling schedule’ [*ϵ*_0_,…,*ϵ*_*T*_]. If the reduction of error demanded between rounds is too great, the algorithm will behave like the rejection sampler. If it is too small, the algorithm may take an extremely long time to run. We propose a novel variant of ABC-SMC that adaptively sets *ϵ*_*t*_ at each round:

(i) initialize *ϵ*_*t*_=0.5*ϵ*_*t*−1_,
(ii) attempt to sample ***θ***_*t*_ from ***θ***_*t*−1_ with error bound *ϵ*_*t*_,
(iii) if (ii) does not succeed within a given number of iterations, define Δ*ϵ*=*ϵ*_*t*_−*ϵ*_*t*−1_,
(iv) terminate if Δ*ϵ*<*τ*. Otherwise, repeat (ii) with $\epsilon_{t}\leftarrow \epsilon_{t-1}+0.5\Delta \epsilon$.

A similar strategy for automated calculation of the cooling schedule is now implemented in the ABC-SysBio Python package [35]. While the SysBio implementation allows selection of an *α* parameter determining the quantile of the previous population to be used for the next error cut-off (arbitrarily initially set to 0.5 in our implementation), the implementation does not adaptively adjust *α* if the population is not filled in a certain number of iterations, unlike in our approach. Our adaptive error shrinking is thus relatively parameter-free, though a carefully chosen *α* (or heuristically determined cooling schedule) would be expected to show faster performance for a specific problem.

Our ABC algorithm maintained a posterior population of 100 particles and performed a maximum of 10 000 draws from the previous estimate before reattempting with a higher error threshold. A ‘no improvement’ threshold was set to cause the algorithm to terminate if successive rounds did not decrease the maximum error by more than 0.003. This cut-off was chosen based on the magnitude of the minimum RMSE attained by a simulated voltage clamp trace under reported parameters (equations (2.11)–(2.16)).

#### 3.2.2 Approximate Bayesian computation settings for fitting of Hodgkin–Huxley parameters

Prior distributions over all parameters were assumed to be independently uniform with width roughly an order of magnitude greater than the reported value. A random walk kernel distributed as a zero-centred normal with variance roughly 10% of the width of the associated prior was applied to each draw. Exact specifications of prior and kernel distributions are detailed in table 1.

**Figure 2.**
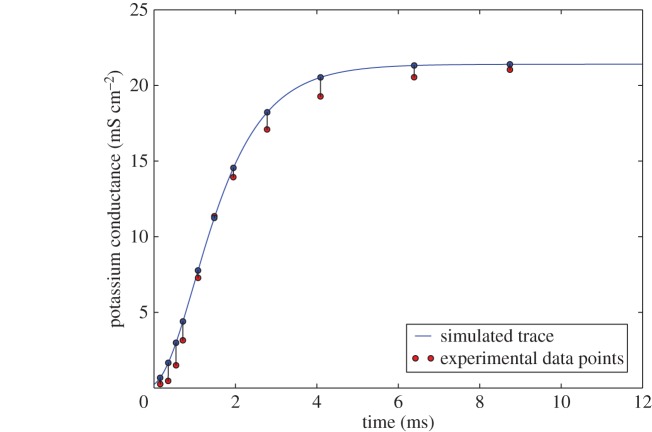
Representation of distance calculation for potassium conductance between experimental data points and data simulated with functional curation for a single voltage clamp experiment (§3.1). Experimental data points are shown in red, while the simulated trace is shown in solid blue, with points corresponding to the time of experimental recordings marked with circles. Distances between these points and their experimental counterparts (shown in black lines) are used by equation (3.1) to calculate the objective function for ABC.

For each voltage clamp experiment, data were digitized from the original Hodgkin and Huxley publication in the form of a pair of vectors: time points, ***t***, and the ionic conductance (either sodium or potassium) at each time point, ***g***(*t*). When fitting the limited form of the Hodgkin–Huxley model (equations (2.7), (2.9) and (2.10)), the experimental data from each voltage clamp were used to fit the six parameters at the experimental depolarization. Thus, the distance function employed by ABC when fitting *α* and *β* was the squared distance between the experimental conductance and the simulated conductance $\hat{g(t)}$ at all reported time points *t*∈[0,*T*] of a given voltage clamp, which is represented visually in figure 2:

3.7 $$D=\sqrt{\frac{1}{T}\sum_{t=0}^{T}{\left(g(t)-\hat{g}(t)\right)}^{2}}.$$

**Figure 3.**
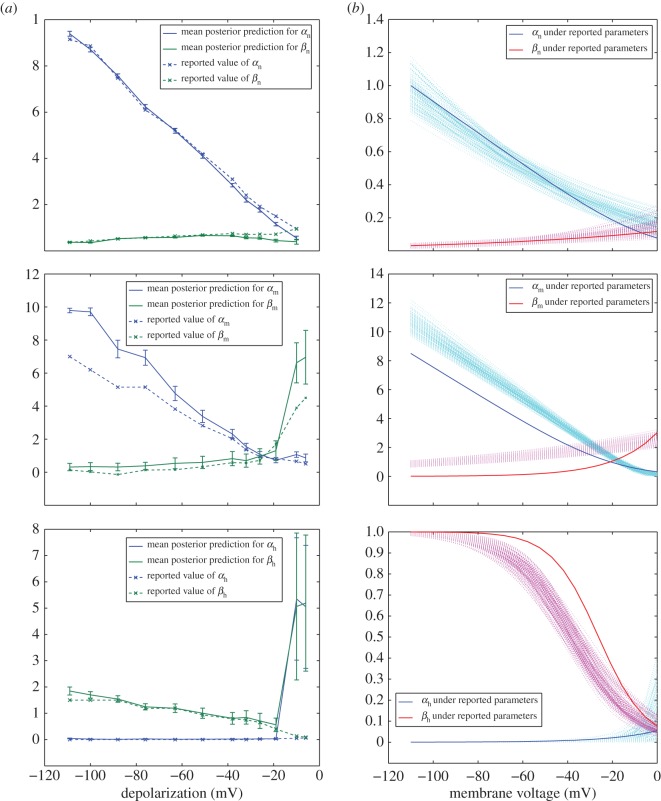
Visualizations of ABC posterior estimates for column (*a*): conductance gating rate parameters (equations (2.7), (2.9) and (2.10)) and column (*b*): voltage dependency parameters (equations (3.1)–(3.6)). In (*a*), ABC mean estimates for *α* and *β* are depicted in solid lines, with error bars indicating 1 s.d. above and below these mean estimates. In (*b*), each particle in the ABC posterior estimate for the voltage dependency parameters is used to generate a trace of *α*, *β* according to equations (3.1)–(3.6) in a dotted line, while traces generated by reported parametrizations are represented by solid lines. Both (*a*,*b*) depict, from top to bottom, (*α*_*n*_, *β*_*n*_), (*α*_*m*_, *β*_*m*_) and (*α*_*h*_, *β*_*h*_).

When fitting the full, voltage-dependent Hodgkin–Huxley model (equations (3.1)–(3.6)), all voltage clamp data were employed at once to capture time- and voltage-dependent effects. This is equivalent to including *M* equally weighted experimental repetitions. Thus, the distance function employed by ABC in this instance is simply the RMSE between the simulated data ${\hat{g}}_{j}(t)$ and the experimental data (defined in equation (3.1)) averaged over all *M* voltage clamp experimental traces ***g***_*j*_ (reported for a single axon preparation in figs 3 and 6 of the original publication):

3.8 $$D=\frac{1}{M}\sum_{j=1}^{M}\sqrt{\frac{1}{T}\sum_{t=0}^{T}{\left(g_{j}(t)-{\hat{g}}_{j}(t)\right)}^{2}}.$$

## 4. Results

### 4.1 Approximate Bayesian computation posteriors on gating rate parameters *α* and *β*

We began with inference on the parameters of the simplified Hodgkin–Huxley model (equations (2.6)–(2.10)), as the data used by the authors to arrive at their reported parametrizations were visually reported in the original publication. Posteriors over the six gating rate parameters (*α*_*n*_, *β*_*n*_, *α*_*m*_, *β*_*m*_, *α*_*h*_ and *β*_*n*_ from equations (2.7), (2.9) and (2.10)) were inferred by ABC using the digitized voltage clamp data as described in §ii. Summaries of the final posterior estimates obtained by applying the adaptive error shrinking implementation of ABC (§i) are shown in figure 3*a*.

In figure 3*a*, we see a high homology between the values of *α*_*n*_ and *β*_*n*_ reported for the data depicted in fig. 3 of the original publication and the mean values of the posterior estimates produced by ABC. We also see universally small standard deviations about the mean posterior estimates, indicating a high degree of confidence that they reflect a well-constrained optimal value. When the magnitude of the depolarization is smaller, we see a slight systematic deviation of the mean posterior estimates produced by ABC from the original reported values. We attribute this to the ability of our automated method to produce a better fit than the manual methods of Hodgkin and Huxley when fluctuations in the data (∝|*min*(*g*_*K*_)−*max*(*g*_*K*_)|) are small. Indeed, we found the RMSE performances of all particles in these posterior estimates exceed that of the reported parametrization (not shown), indicating an improvement in fit to the experimental data by the ABC estimates.

Figure 3*a* shows a less perfect homology between ABC mean posterior values of *α*_*m*_,*β*_*m*_,*α*_*h*_,*β*_*h*_ and those reported by Hodgkin and Huxley, as well as generally larger standard deviations, which is not unexpected given the increased complexity of the function for sodium conductance (equation (2.8)). For the activation term *m*, which controls the initial increase of sodium conductance, we see that the ‘spike’ parameter *α*_*m*_ shows lower homology with reported values for high depolarizations (as well as looser bounds) yet traces from the final ABC estimates were found to achieve better RMSE performance than traces with reported values. The higher uncertainty at large depolarizations despite improvement of fit may be explained by the low frequency of sodium conductance sampling during the AP in fig. 6 of [6], which results in very few data points falling on the spike itself. Poor definition of this portion of the curve would lead to multiple, equally fit choices for *α*_*m*_, and thus a larger variance in the ABC posterior. ABC mean values for the ‘plateau’ parameter *β*_*m*_ are reasonably consistent with reported values with some exception towards low depolarizations, where the similar RMSE performance and loose bounds may indicate a non-unique parametrization for the complex function, resulting from low deviation in *g*_*Na*_ for |Δ*V* |<10 *mV*.

For the inactivation term *h*, ABC posterior estimates for *α*_*h*_ and *β*_*h*_ are both tightly bounded and show high mean homology with reported values (particularly *α*_*h*_, which is effectively 0 throughout) except for low-magnitude depolarizations. At |Δ*V* |<10 *mV*, however, the sodium conductance inactivation gate *h* is effectively irrelevant, as the conductance never reaches a significant spike in activation. At these depolarization values, the shape of the sodium conductance curve begins to resemble that of potassium conductance, the model for which contains no decay term. This lack of impact of *h* on the observable quantity *g*_*Na*_ explains the large variations of the posteriors around *α*_*h*_ and *β*_*h*_.

### 4.2 Approximate Bayesian computation posteriors on voltage dependency parameters *k*

After fitting the *α* and *β* parameters describing the gating rates for potassium and sodium current conductance, we attempted to fit the parameters of the full Hodgkin–Huxley model (equations (3.1)–(3.6)), which describe the voltage-dependency for the said rates. The forms of these functions were empirically chosen by the authors to fit the variation of their estimates of the *α* and *β* parameters of the simplified model over a range of depolarization values. These *α* and *β* values were in turn inferred from the shape of the ionic conductance data described in figs 3 and 6 of the original publication and represented as dashed lines in figure 3*a*. Rather than this indirect means of fitting—first deriving values of the rate constants and then parameters for the functions thought to describe them—we sought to fit the parameters of equations (3.1)–(3.6) directly to the experimental ionic conductance data (using the distance function described in equation (3.2)). This effectively expanded the parameter space for ABC inference from the six in the previous section to 14, but also expands the training dataset from one to 12 traces.

Summaries of the final ABC posterior estimates for the potassium conductance parameters are reported in table 2. We initially note that the mean posterior estimates of *k*_*α*_*n*_2_ and *k*_*α*_*n*_3_ demonstrate high deviation from the reported values. Additionally, we find the reported values of these parameters lie outside the 90-percentile range around this mean. The significance of this deviation, however, is negated by the large spread of posterior estimates for these parameters, quantified both by the variance about the mean and the width of the 90-percentile range, which suggests a lack of confidence in the mean posterior estimate. Similarly, while the reported value of *k*_*β*_*n*_2_ lies within the 90-percentile range about the mean posterior estimate, the width of the distribution suggests the ‘recovered’ estimate is not unique. The width of the posterior estimates of these three parameters thus suggests an unidentifiability in equations (3.1) and (3.2).

**Figure 4.**
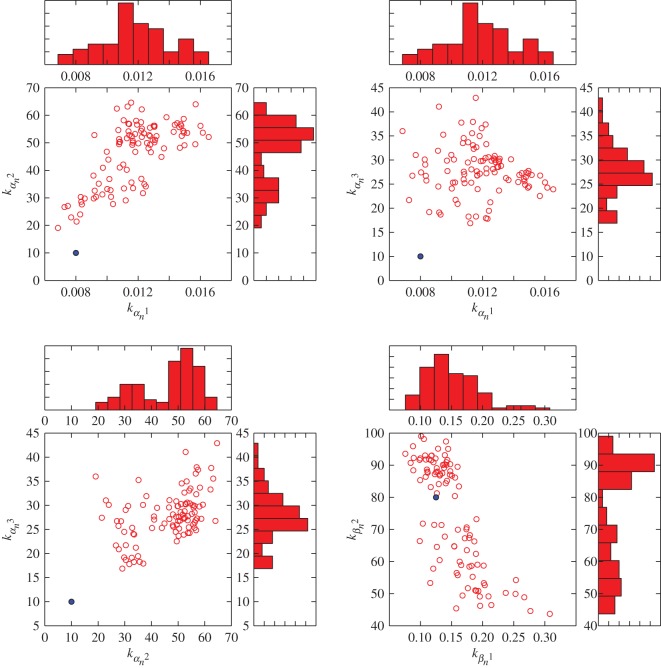
Biplot visualization of relationships between voltage dependency parameters for potassium conductance activation (equations (3.1) and (3.2)) in an ABC posterior estimate. In each plot, red circles indicate a particle from the ABC posterior estimate, while the solid blue dot indicates the reported value's location in parameter space. Histograms along each axis indicate the marginal frequencies for each value of the parameter.

As the three highly variable parameters *k*_*α*_*n*_2_, *k*_*α*_*n*_3_ and *k*_*β*_*n*_2_ are all contained in the exponential portions of the equations, we sought to determine whether this could be a structural unidentifiability caused by the choice of an overly complex functional form. In figure 3*b*, we parametrized equations (3.1) and (3.2) according to each particle in the ABC posterior estimate and plotted the resulting values of *α*_*n*_ and *β*_*n*_ over a range of membrane voltages. In the case of a structural unidentifiability, we would expect low variation in the function output despite high variation in parameter space. Instead, we observe a relatively wide distribution around *α*_*n*_ at all values of *V* , and a widening distribution around *β*_*n*_ at low values of *V* when *k*_*β*_*n*__ begins to dominate the exponential portion of the function. This suggests that fluctuations in parameter values lead to commensurate fluctuations in the observable output. Additionally, the biplot of the posterior estimate in figure 4 fails to reveal any strong correlations between parameters that might be expected of a structural unidentifiability. The marginal distributions of the highly variable parameters *k*_*α*_*n*_2_, *k*_*α*_*n*_3_ and *k*_*β*_*n*_2_ also show one or more distinct peaks, which indicate preference of the algorithm for certain values despite the overall uncertainty. This would not be expected if fluctuations in these parameters did not affect the observable quantity *g*_K_. Together, this suggests that the full Hodgkin–Huxley model for potassium conductance exhibits a practical, rather than structural, unidentifiability.

ABC posterior estimates for sodium conductance voltage dependency parameters are comparatively well constrained. While table 3 shows several parameters with high mean deviation from reported values (*k*_*α*_*m*_2_, *k*_*α*_*m*_3_, *k*_*β*_*m*_2_ and *k*_*α*_*h*_2_), posterior estimates for all parameters show low relative variance and 90-percentile spread with the exception of *k*_*α*_*h*_2_. This, coupled with the substantial decrease in RMSE of all particles in the posterior when compared to the model under reported parametrization (indicated in the bottom row of table 3), suggests that ABC has arrived at a relatively tightly bounded optimum exceeding that of the manual methods of the original paper. Figure 3*b* seems to support this, as posterior traces around *α*_*m*_, *β*_*m*_ and *β*_*h*_ notably deviate from the reported traces yet maintain a constrained form across all values of *V*.

**Table 3.** Summary statistics of ABC posterior estimates for voltage dependency parameters of sodium conductance gating rates. (Parameter names in the leftmost column refer to the corresponding labels in equations (3.3)–(3.6). ‘Mean’ and ‘variance’ columns indicate the summary statistics used to describe the final posterior estimate of ABC, while ‘5th, 95th percentiles’ denote the points at which the posterior CDF records 5% and 95% density, respectively (analogous to 90% CI). The ‘RMSE’ row contains the value of the distance metric (equation (3.2)) attained by the reported parameters, as well as the minimum and maximum (bounded from above by the final ABC error constraint *ϵ*_*T*_) values attained by particles in the final posterior estimate.)

		ABC posterior estimates		
parameter	reported value	mean	variance	5th, 95th percentiles
*k*_*α*_*m*_1_	0.1	0.110	3.69×10^−5^	0.102, 0.121
*k*_*α*_*m*_2_	25	13.3	3.43	10.8, 16.1
*k*_*α*_*m*_3_	10	3.73	1.36	1.68, 5.66
*k*_*β*_*m*_1_	4	2.59	0.0915	2.17, 3.12
*k*_*β*_*m*_2_	18	95.9	9.87	91.1, 99.6
*k*_*α*_*h*_1_	0.07	0.414	0.0978	0.0315, 0.917
*k*_*α*_*h*_2_	20	5.73	17.3	1.08, 13.0
*k*_*β*_*h*_1_	30	40.6	8.74	36.8, 45.6
*k*_*β*_*h*_2_	10	13.6	3.01	11.5, 16.6
RMSE	1.93	min 1.20, max (≤*ϵ*_*T*_) 1.22		

**Figure 5.**
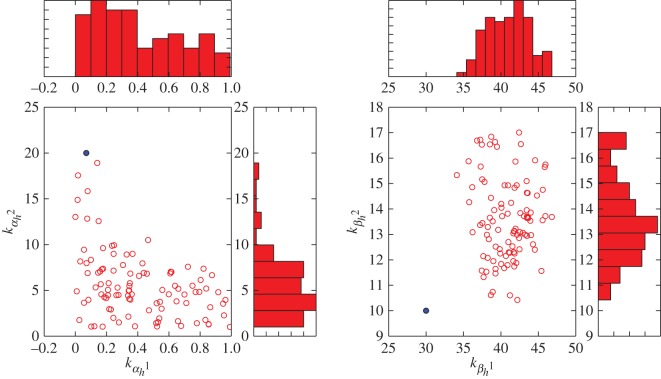
Biplot visualization of relationships between voltage dependency parameters for sodium conductance inactivation (equations (3.1) and (3.2)) in an ABC posterior estimate. In each plot, red circles indicate a particle from the ABC posterior estimate, while the solid blue dot indicates the reported value's location in parameter space. Histograms along each axis indicate the marginal frequencies for each value of the parameter.

In figure 3*b*, *α*_*h*_ shows high input sensitivity to variation in the unconstrained parameter *k*_*α*_*h*_2_ at low values of *V* , where *k*_*α*_*h*_2_ dominates the exponential portion of equation (3.5). This is not suggestive of structural unidentifiability, as fluctuation in the posterior distribution is manifest in the observable quantity *α*_*h*_. A biplot visualization of the ABC posterior (figure 5) fails to show any correlation between the voltage dependency parameters of *h*, and the marginal distribution of *k*_*α*_*h*_2_ has clear skew, indicating the ability of the algorithm to discern and penalize fluctuations in its value. This supports classification of *k*_*α*_*h*_2_ as a practically, rather than structurally, unidentifiable parameter.

### 4.3 Posterior performance on voltage protocols

To investigate experimental designs that could be employed to further constrain the ambiguous parameters returned from our ABC analysis (or, failing that, support classification of said variation as inherently biological), we investigated the emergent behaviour of the parametrized models when subjected to the four voltage protocols proposed by Hodgkin and Huxley in figs 20–23 of their original publication. Under these protocols, Hodgkin and Huxley's numerical solutions to equations (2.11)–(2.16) showed general qualitative agreement with experimental behaviour [6]. Two of these protocols were designed to probe the ability of the cell to respond to membrane potential injections of variable magnitude or timing. The ‘positive phase depolarization’ protocol assessed the degree to which a voltage stimulus during the recovery phase of the AP could trigger a secondary activation, while the ‘sub-threshold depolarization’ experiment sought to assess behaviour of the membrane potential under depolarizations insufficient to trigger a full AP. The other two protocols, ‘anode break excitation’ and ‘oscillation induction’, were designed to probe the behaviour of the membrane under new clamping conditions. The anode break protocol introduced an anodal polarization, lowering potassium conductance activation and sodium conductance inactivation, reversing the membrane current at resting potential and causing full excitation after release of the clamp. The oscillation induction experiment probed the observed oscillation of the membrane potential in response to small, sustained current injections.

For each experiment, either the sodium or potassium conductance model was allowed to vary according to the ABC posterior estimate (described in §4.2), while the other was held to default values. Simulating the membrane voltage under these conditions allowed us to attribute differences from reported behaviour to variation in the behaviour of a single channel, and thus assess the ability of the protocol to further constrain the conductance models.

#### 4.3.1 Variable behaviour of potassium posterior

**Figure 6.**
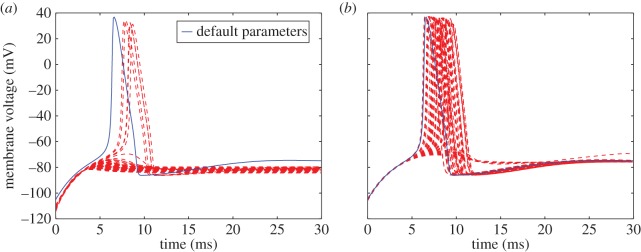
Membrane voltage response to the anode break excitation protocol. Models parametrized according to the posterior estimates for potassium (*a*) or sodium (*b*) conductance parameters of equations (3.1)–(3.6) are shown in red, whereas response under default parameters is shown in blue.

Under the anode break excitation experimental protocol, the model's membrane voltage showed variable behaviour over the potassium posterior ranging from total inactivation to full excitation nearly matching the experimental trace in both magnitude and temporal placement (figure 6*a*). This suggests that the experiment might be informative for further constraining one or more parameters that showed high posterior variation under voltage clamp data alone, which is not entirely surprising given that the hyperpolarizing current alters the potassium conductance at resting potential, potentially revealing ion channel kinetics that were absent under the simpler protocols.

**Figure 7.**
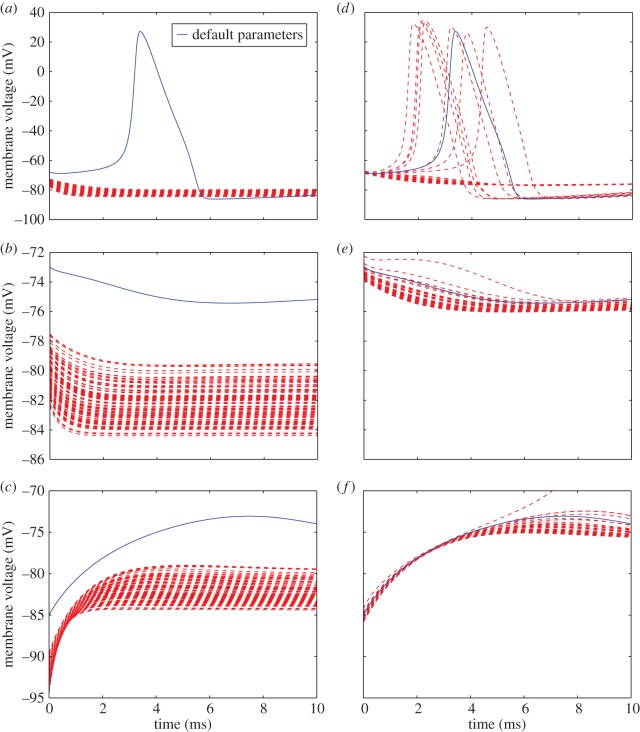
Membrane voltage response to the threshold depolarization protocol for (from top to bottom) 7 mV, 2 mV, −10 *mV*. Models parametrized according to the posterior estimates for either potassium (*a*–*c*) or sodium (*d*–*f*) conductance parameters of equations (3.1)–(3.6) are shown in red, whereas response under default parameters is shown in blue.

**Figure 8.**
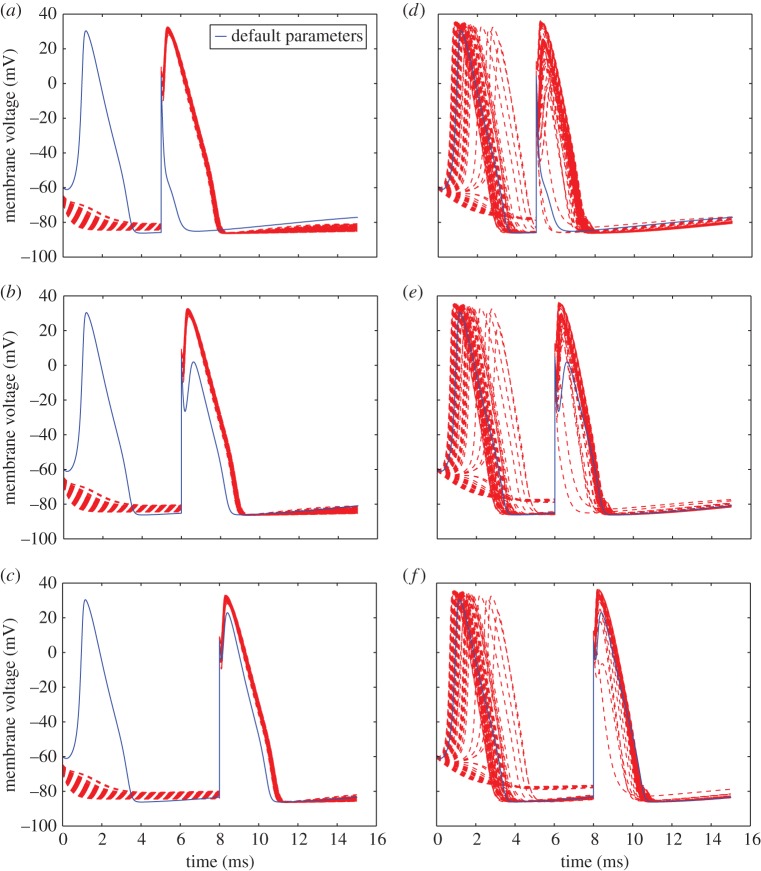
Membrane voltage response to the positive phase depolarization protocol at (from top to bottom) 5 ms, 6 ms, 8 ms. Models parametrized according to the posterior estimates for either potassium (*a*–*c*) or sodium (*d*–*f*) conductance parameters of equations (3.1)–(3.2) are shown in red, whereas response under default parameters is shown in blue.

**Figure 9.**
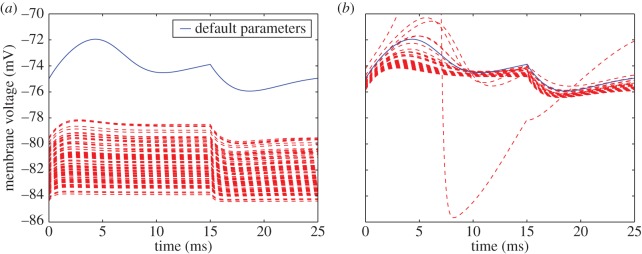
Membrane voltage response to the oscillation induction protocol. Models parametrized according to the posterior estimates for potassium (*a*) or sodium (*b*) conductance parameters of equations (3.1)–(3.6) are shown in red, whereas response under default parameters is shown in blue.

Both the positive phase depolarization experiment and the 7 mV (figure 7*a*) threshold excitation experiment reveal the inability of the posterior parametrized models to trigger an AP in response to the same membrane stimulus sufficient for the reported parametrization. The positive phase depolarization experiments (figure 8*a*–*c*) also show a more exaggerated second activation response to a stimulation during restitution across all posterior parametrized models. At low depolarization (figure 7*b*) or slight polarization (figure 7*c*) of the membrane, as well as the oscillation induction experiment (figure 9*a*), the posterior parametrized models showed the same qualitative behaviour, yet at a notable downward shift in voltage.

This inability to recreate the reported behaviour may be owing to the fact that the leakage current component of the model (*I*_*l*_ in equation (2.2)) was set by the authors after fitting the sodium and potassium conductances as a ‘catch-all’ to ensure matching to experimental behaviour. As such, this value is probably not a biological truth, and employing it without a similar adjustment for our parametrizations may lead to this failure to capture the same behaviour. Regardless, it appears from these results that separately fitting the potassium conductance parameters to the voltage clamp experiments with ABC failed to produce a parametrized model capable of exhibiting all of the behaviour reported by the authors.

#### 4.3.2 Variable behaviour of sodium posterior

Under the anode break excitation experimental protocol, the qualitative behaviour under-reported values is captured nearly universally across all posterior parametrized sodium conductance models, with variation largely constrained to the temporal placement of the excitation spike (figure 6*b*). This suggests that information may be gained from this protocol as to the unidentifiable parameter *k*_*α*_*h*_2_, which as in the case of the potassium *n* gate parameters is unsurprising, given that the hyperpolarization of the membrane also decreases sodium current inactivation (controlled by *h*), leading to the reversal of membrane current and potentially exposing new ion channel kinetics.

Unlike the behaviour of the models parametrized under the potassium posterior, at least some models parametrized under the sodium posterior capture the membrane potential behaviour after nearly any depolarization (figure 7*d*–*f*). We additionally see a range of behaviour, including the reported behaviour, when examining AP activation during both the first and second depolarizations of the positive phase depolarization experiment (figure 8*d*–*f*). Interestingly, and again unlike the potassium posterior, we see that a particle in the posterior has a large outlier with regard to response to the current clamp experiment, and thus even the well-constrained model is sensitive to fluctuations within its estimates (figure 9*b*). We may conclude that some further information on the parameters of the model could be gleaned by examining model response to any of these protocols as well as that of the anode break experiment.

## 5. Discussion

ABC posterior estimates for the parameters of the simplified Hodgkin–Huxley model were tightly constrained across most magnitudes of depolarization, indicating that the model was identifiable from the data under most experimental conditions. Not only this, but the mean estimates showed high homology with the values reported by Hodgkin and Huxley in their original publication. Such a well-constrained recovery of the original parameters highlights the remarkable degree of accuracy achieved by the authors without the aid of modern computational tools. Even when the posterior estimates of the ABC approach widened at low magnitude depolarizations, particularly around the parameters controlling the sodium inactivation gate *h*, simulations employing the estimates of the original authors were able to reproduce the experimental data.

Performing ABC parameter inference on the full Hodgkin–Huxley model highlighted the value of the method's ability to quantify model uncertainty. The wide posteriors around certain parameters in the potassium (*k*_*α*_*n*_2_, *k*_*α*_*n*_3_ and *k*_*β*_*n*_2_) and sodium (*k*_*α*_*h*_2_) conductance components suggested an inability to fully fit the model under the provided experimental data. Because the output of ABC is the full population estimating the posterior over the parameters, we were able to take advantage of additional information, such as pairwise correlations and marginal distributions over the parameters, to support classification of the unidentifiabilities as practical rather than structural. This analysis suggests that additional data may be useful in further constraining the model, although without further experimentation we cannot rule out the unidentifiability being attributed to natural biological variability. In either case, this study highlights the usefulness of ABC in reporting all relevant statistics about the posterior within the final estimating population.

The classification of the model unidentifiability as practical rather than structural suggests that Hodgkin and Huxley arrived at a model of the appropriate degree of complexity to describe their system. This also suggests that the authors could not have found a better parametrization of their model without the inclusion of additional data that could further inform the ion channel dynamics within the model. Despite this, we do observe instances where the ABC posterior estimates show notable performance gains over the reported model parametrization (table 3). This is probably a result of our fitting to data from a single recording of a single axon, whereas the authors employed unreported data from several different axons, which would be expected to exhibit variation in their conductance recordings. Using only the single-recording data available to us, it would be unwise to conclude anything significant from the deviations in mean posterior estimates and resulting gains in RMSE performance for particles in the posterior. Instead, the value of our approach lies in the quantification of confidence around the mean parameter estimates given just a sample of the data that was available to the original authors.

To assess what the original authors might have been able to accomplish with the experimental data for the protocols they proposed at the end of their paper (and armed with modern computational tools), we examined the differences in response to several complex protocols over the particles in the posterior estimates for both potassium and sodium conductance. We noted that the particles of the sodium conductance posterior showed greater consistency in the response to these protocols, both internally and when compared to the response of the reported parametrization, than the particles of the potassium posterior. This is probably owing to the presence of higher posterior variability in the potassium conductance model. The failure of all particles in the posterior to produce the same behaviour as the reported parametrization could be indicative of dependencies between the parameters of the two conductance models not captured by the separate fitting approach, or simply another constraint on the model not captured by the voltage clamp protocol.

While there appears to be additional information that could be leveraged from these experiments to further constrain both conductance models, the leveraging of this information to potentially decrease model uncertainty would be non-trivial. Each of these experimental protocols, including the standard voltage clamp, will only constrain a subset of the model parameters. Thus, fully constraining, the model would require leveraging a weighted combination of the deviation from experimental data under each protocol in our distance function. Such a weighted combination of data introduce so-called ‘hyperparameters’ into the ABC algorithm—parameters that control how much importance the algorithm gives to each source of data. These parameters are difficult to assign, especially without knowledge of the precision of the measurements being included, and might require an additional level of parameter fitting to set. Given the sparsity of the experimental data, it is unlikely that these hyperparameters could be reliably constrained. Even using the posteriors from the voltage clamp ABC as priors for a new round of ABC employing more complex voltage protocols would require a weight to be assigned for the trade-off between prior likelihood and experimental performance. This amounts to attempting to maintain the behaviour of the model under the voltage clamp protocol while simultaneously seeking to improve performance under the new protocol. In light of these limitations, we believe full integration of simulated data from these protocols to be beyond the scope of the paper. We can only theorize as to the degree of constraint they could have produced on the model, or the support they would lend to classifying the uncertainty as inherent biological variation by failing to further constrain it.

Arriving at an optimal means to combine data from multiple protocols, or simply determining a protocol that provides the most information on a given model, are problems of ‘experimental design’, and will be a focus of future work with ABC parameter fitting techniques, owing to its close relationship to model identifiability.

## 6. Conclusion

ABC parameter fitting of the Hodgkin and Huxley model to the voltage clamp data reported in their original paper was able to recover precisely the rate parameters controlling sodium and potassium channel gating, but was less able to recover the parameters controlling the voltage dependency of said rates. The width of the distributions around these unidentifiable parameters, as well as their lack of correlation with each other and the relative sensitivity of the output to their fluctuations, suggested that these were practically unidentifiable, potentially requiring more data to constrain. Investigation of differential behaviour under more complex voltage protocols thought to probe the conductance dynamics more fully revealed several promising sources of further model constraint.

We hope that this study serves as both a strong affirmation of the quality of the original work done by Hodgkin and Huxley to fit their model, which appears to be near-optimal under the data available to them, as well as a template for similar identifiability analyses in current cardiac models. While we doubt that many present-day models will be able to be constrained as effectively as the Hodgkin–Huxley model, given the increased complexity and the myriad sources of data, we believe the uncertainty quantification provided by these analyses can pinpoint directions for future experimental work, or provide insight into the degree and distribution of biological variability in the system. The adoption of these Bayesian parameter fitting methods would foster a documented link between experiment and data that has slowly been lost since the original work done by the founders of the field of computational biology, Hodgkin and Huxley.

## Data accessibility

A repository containing the digitized Hodgkin–Huxley data, CellML files, ABC implementation, functional curation protocols and Python scripts used to generate the results presented in this paper are freely available to the public in the Paper Tutorials section of the Chaste wiki (https://chaste.cs.ox.ac.uk/trac/wiki/PaperTutorials/HodgkinHuxleyABC), along with instructions on how to compile and execute the code, or by browsing the source code directly (https://chaste.cs.ox.ac.uk/trac/browser/projects/HodgkinHuxleyABC).
